# Role of AMPK and Sirtuins in Aging Heart: Basic and Translational Aspects

**DOI:** 10.14336/AD.2024.1216

**Published:** 2025-11-13

**Authors:** Maria Luisa Barcena, Muhammad Aslam, Kristina Norman, Christiane Ott, Yury Ladilov

**Affiliations:** ^1^Department of Urology, Eberhard Karl University of Tuebingen, Tuebingen, Germany.; ^2^Experimental Cardiology, Department of Internal Medicine I, Justus Liebig University, Giessen, Germany.; ^3^Department of Cardiology, Kerckhoff Clinic GmbH, Bad Nauheim, Germany.; ^4^DZHK (German Centre for Cardiovascular Research), Partner Site Rhein-Main, Bad Nauheim, Germany.; ^5^Charité - Universitätsmedizin Berlin, corporate member of Freie Universität Berlin, Humboldt-Univeristät zu Berlin, and Berlin Institute of Health, Department of Geriatrics and Medical Gerontology, Berlin, Germany.; ^6^Department of Nutrition and Gerontology, German Institute of Human Nutrition Potsdam-Rehbrücke, Nuthetal, Germany.; ^7^Institute of Nutritional Science, University of Potsdam, 14558 Nuthetal, Germany.; ^8^DZHK (German Centre for Cardiovascular Research), Partner Site Berlin, Berlin, Germany.; ^9^Department of Cardiovascular Surgery, Heart Center Brandenburg, University Hospital, Brandenburg Medical School Theodor Fontane, Bernau, Germany.

**Keywords:** aging, metabolic sensing, AMPK, sirtuins, heart failure, vascular diseases, inflammation, microbiome

## Abstract

Aging is a key risk factor for numerous diseases, including cardiac diseases. High energy demands of the heart require precise cellular energy sensing to prevent metabolic stress. AMPK and sirtuins are key intracellular metabolic sensors regulating numerous cell functions, like mitochondrial function and biogenesis, autophagy, and redox balance. However, their function is impaired during the aging process leading to mitochondrial dysfunction, oxidative stress, and inflammation culminating in cardiovascular diseases. The underlying molecular mechanisms leading to dysfunction of metabolic sensing in the aging heart are complex and comprise both intracellular and systemic age-related alterations. In this study, we overview the current knowledge on the impact of aging on cardiac metabolic sensing, with a focus on AMPK and sirtuins, while mTOR pathway was only marginally considered. A particular focus was given to systemic factors, e.g., inflammation, vascular diseases, and microbiome.

## 1. Basic aspects

### 1.1 Effect of aging on metabolic sensing through AMPK and sirtuins

#### Regulation

The 5’ AMP-activated protein kinase (AMPK) is a crucial metabolic sensor and regulator in mammalian cells playing a key role in cellular energy balance. Activation of AMPK stimulates catabolic processes while suppressing anabolic processes to restore energy balance [1, 2].

Mammalian AMPK is a heterotrimeric complex comprising of a catalytic α-subunit and two regulatory β- and γ-subunits. The γ-subunit contains binding sites for AMP, ADP, and ATP [3, 4], whose occupancy depends upon the cellular AMP:ATP and ADP:ATP ratios. In energy-depleted conditions, characterized by high AMP:ATP ratio, ATP bound to the γ-subunit is replaced by AMP, resulting in allosteric modification of AMPK that allows access of upstream kinases to the key phosphorylation site at the α-subunit’s Thr172, while reducing access of Thr172 to phosphatases. This leads to increased AMPK phosphorylation and activation. Although AMP is the primary regulator of AMPK activity, ADP may also exert a stimulatory effect [5].

Three upstream kinases directly phosphorylate AMPK at Thr172: liver kinase B1 (LKB1), Ca^2+^/calmodulin-dependent protein kinase kinase 2 (CaMKK2), and TGF-β-activated kinase 1 (TAK1) [6-8]. Phosphorylation of Thr172 can be reversed by protein phosphatase 1 (PP1), PP2A, and PP2C, resulting in the inhibition of AMPK. Moreover, several upstream Ser/Thr kinases, including PKA, AKT, and ERK1/2, can deactivate AMPK through direct phosphorylation of α-subunit (for a review see [9, 10]).

Other key cellular metabolic regulators include the class III histone deacetylases sirtuins (Sirt1-7), which utilize nicotinamide adenine dinucleotide (NAD^+^) as a co-substrate for the removal of acetyl groups from lysine residues of target proteins. However, Sirt4 and Sirt6 primarily function as mono-ADP-ribosyltransferases. Sirtuins regulate a wide variety of biological processes such as metabolism, mitochondrial homeostasis, genomic stability, and redox homeostasis (for review see [11]). Protein deacetylation mediated by sirtuins promotes enzyme-substrate binding and, consequently, enhances enzyme activity [12].

Increasing evidence suggests that AMPK and sirtuins directly interact and cooperate in cellular metabolic regulation. Activation of AMPK leads to the elevation of cytosolic NAD^+^ levels which in turn enhances the activity of sirtuins. This AMPK-dependent NAD^+^ elevation may result from the upregulation of nicotinamide phosphoribosyltransferase (NAMPT), an enzyme involved in NAD^+^ biosynthesis [13] or the activation of mitochondrial β-oxidation [14]. Additionally, AMPK activation may also induce transcriptional upregulation of sirtuins, such as Sirt1 [15]. Reciprocally, Sirt1 may promote AMPK activity by deacetylating and activating AMPK’s upstream kinase LKB1 [16].

#### Effect of aging

Aging significantly impacts the activity and expression of AMPK and sirtuins. Particularly, basal cardiac AMPK activity decreases with age, while AMPK content remains relatively unchanged [10, 17] The precise molecular mechanisms underlying age-related AMPK inactivation are still poorly understood, but disturbance in Ca^2+^ homeostasis and altered activity of upstream phosphatases and kinases, such as PKA and ERK1/2 have been suggested as potential factors [10]. Aging also leads to reduced expression and activity of sirtuins in various tissues, including the heart [18, 19] and vessels [20], which may further contribute to the downregulation of AMPK signaling. Notably, we have recently shown that the myocardial expression of key deacetylases Sirt1 and Sirt3 is significantly reduced in apparently healthy older women but not older men, suggesting a sex-dependent effect of cardiac aging [17]. This aging-related decline in sirtuins expression may be exacerbated by impaired sirtuins activity due to reduced availability of NAD^+^ [21, 22] resulting from increased NAD^+^-consumption. Particularly, increased expression of the NAD^+^-consuming NADase CD38 in various tissues of old mice and humans has been reported [23]. Although the mechanisms underlying CD38 upregulation in aging are not fully understood, inflammation has been proposed to play a role [24]. Hence, chronic systemic inflammation associated with aging (inflammaging) may contribute to CD38 upregulation and subsequent NAD^+^ decline. Notably, genetic deletion of NLRP3 in mice prevented many age-associated changes in the heart and was associated with increased NAD^+^ [25].

Another mechanism leading to reduced NAD^+^ levels is the elevated activity of poly(ADP-ribose) polymerase 1 (PARP1), a key cellular DNA damage sensor. The increased rate of DNA-damage associated with aging may elevate PARP1 activity and NAD^+^ consumption [22].

The reduced expression and/or activity of AMPK and sirtuins negatively affect the cellular energy balance, particularly by disrupting mitochondrial homeostasis [26]. Consequently, the release of reactive oxygen species (ROS) and mitochondrial DNA (mtDNA) from damaged mitochondria promotes cardiac inflammation [27], further exacerbating the adverse effects of aging.

Collectively, mounting evidence argues that the aging-related impairment of metabolic sensing through AMPK and sirtuins contributes to disrupted mitochondrial homeostasis, oxidative stress, and inflammation, ultimately leading to a reduction in health span and progression of diseases.

### 1.2 Role of sirtuins and AMPK in aging effects on mitochondria, ROS, and autophagy

#### Mitochondria

Mitochondria are now recognized not only as bioenergetics centers but also as crucial hubs for intracellular signaling, regulating various cellular processes. Aging in model organisms and humans is associated with a decline in mitochondrial function, particularly in high-energy-demanding organs like the heart, leading to structural disruptions, altered energy production, and impaired mitochondrial signaling [28-31]. This decline in mitochondrial function results in the accumulation of dysfunctional mitochondria, mainly due to the dysregulated quality control processes [32-34]. Consequently, cardiac performance deteriorates due to compromised mitochondrial function and elevated ROS production [35]. Mitochondrial antioxidants like superoxide dismutase 2 (SOD2) are overwhelmed by ROS, leading to oxidative stress damage and activation of pro-inflammatory pathways such as nuclear factor (NF)-κB signaling [36, 37]. Accumulating evidence suggests that the activity of mitochondrial SOD2 is strongly regulated by its acetylation at multiple conserved lysine residues [38]. Among the seven mammalian sirtuins, Sirt3, Sirt4, and Sirt5 are primarily located in the mitochondria and play crucial roles in regulating mitochondrial function. Besides protein deacetylation and ADP-ribosylation, these mitochondrial sirtuins also function as succinyltransferases and malonyltransferases [39, 40]. The major mitochondrial deacetylase Sirt3 has been linked to modulating mitochondrial metabolism and lifespan extension in experimental animals. Its anti-aging effects appear to be conserved across different organisms, from yeast to humans. By activating mitochondrial complex I subunits and enzymes in the tricarboxylic acid (TCA) cycle, Sirt3 enhances oxidative phosphorylation and ATP production [41, 42]. Studies with Sirt3 knockout cells demonstrate reduced ATP levels, which are restored upon reintroducing functional Sirt3 [43]. Muscles from mice lacking Sirt3 exhibit decreased oxygen consumption, increased ROS production, and higher oxidative stress [44]. Cell culture studies also confirm that cells lacking Sirt3 have elevated ROS levels, leading to DNA damage and activation of HIF-1α [45]. Additionally, Sirt3 prevents mPTP formation in cardiac muscle by deacetylating cyclophilin D [46]. Sirt3 knockout animals develop cardiac hypertrophy and exhibit higher mortality rates in cardiac stress models [46]. Furthermore, aging results in reduction of mitochondrial Sirt3 expression leading to downregulation of PGC-1α and SOD2. Therefore, aging mitochondria are unable to effectively remove ROS [47, 48]. Interestingly, the detrimental effects of Sirt3-loss on life span could be mitigated by caloric restriction, suggesting a complex interplay between Sirt3 activity, oxidative stress, and longevity [49]. The authors of this study found that Sirt3 deficiency in male mice decreased oxidative metabolism capacity and ATP production, yet significantly extended their lifespan under caloric restriction compared to wild-type mice. This surprising effect of Sirt3 deletion combined with caloric restriction may be due to reduced spontaneous activity or changes in substrate utilization.

AMPK plays a key role in maintaining mitochondrial homeostasis by regulating energy balance and mitochondrial biogenesis [50]. AMPK activates pathways that enhance mitochondrial function and energy production, including the upregulation of PGC-1α [51, 52], a nodal regulator of mitochondrial biogenesis [53]. Additionally, AMPK promotes the removal of damaged mitochondria through mitophagy, ensuring a healthy mitochondrial population [50, 54]. By sensing cellular energy levels and responding to metabolic stress, AMPK helps maintain the integrity and efficiency of the mitochondrial network [50], which is essential for cellular health and function.

#### ROS

The generation of ROS is a crucial mechanism for maintaining physiological cellular processes and is finely regulated by the balanced activities of various antioxidant enzymes [55]. However, an imbalance between ROS production and antioxidant mechanisms may lead to oxidative stress due to the accumulation of free radicals resulting in cellular damage (reviewed in [56]). ROS can reversibly modify redox-sensitive proteins such as Nrf2 and NF-κB, thereby regulating their activity [36, 57]. The main sources of cellular ROS in the aging heart are NADPH oxidases (NOXs) and the mitochondrial electron transport chain (ETC) [58]. ROS generated by NOXs are implicated in hypertension, atherosclerosis, and endothelial dysfunction related to diabetes and aging [59]. NOX2 plays a crucial role in age-associated cardiac remodeling by promoting matrix metalloproteinase activation, pro-fibrotic factor expression, and cardiomyocyte hypertrophy [60]. Likewise, NOX4 is upregulated and is a continuous source of ROS in the aging heart [61].

Nitric oxide (NO) generated by NO synthase (NOS) is essential for normal cardiac function and is protective in ischemic and failing heart. Aging and cardiovascular disease result in reduced NO production and increased formation of toxic peroxynitrite [62], which can inhibit sirtuins [63], leading to enhanced protein acetylation and dysregulation of their activity. Loss of NOS1 or NOS3 results in cardiac hypertrophy in mice and NOS1/NOS3 double knockouts develop age-related cardiovascular phenotype (e.g. hypertension and hypertrophy) [64].

Mitochondrial ETC complexes I and III are the main source of free radicals generated by mitochondria. The major components of the mitochondrial antioxidant system include SOD2, catalase, and glutathione peroxidase. Direct evidence links mitochondrial ROS to cardiac aging: mice with mitochondrial-targeted catalase (mCAT) overexpression live longer and exhibit reduced mitochondrial oxidative stress, mtDNA mutations, and enhanced cardiac function [65, 66]. ROS can directly oxidize cysteine residues of Sirt1, thereby inducing its inactivation [67]. This Sirt1 inhibition may be relevant for the aging heart since Sirt1 expression and activity are reduced in aged hearts accompanied by increased ROS [68, 69]. Conversely, overexpression of Sirt1 in mouse hearts protects from oxidative stress and age-related cardiac hypertrophy [70]. The effects of mitochondrial ROS also vary with age. In young mice, reducing mitochondrial ROS levels does not confer benefit and may even mimic the cardiac proteome of older animals [71], suggesting that lowering already low levels of ROS could be harmful. On the other hand, in aged mice, overexpression of mitochondrial catalase (which scavenges ROS) or mitochondria-specific ROS scavenger has beneficial effects [72], indicating that moderate ROS reduction may be protective in aged hearts [73].

Sirt1 deacetylates AMPK-activating enzyme LKB1 and transcription coactivator PGC-1α thus promoting mitochondrial biogenesis [16, 74]. Consequently, AMPK activation supports sirtuins activity and enhances the synthesis of antioxidant enzymes, which in turn limit ROS production [75]. Sirt3 plays a crucial role in controlling mitochondrial deacetylation, impacting multiple proteins in the ETC to maintain ATP levels and redox homeostasis [76, 77]. Sirt3 deficiency leads to increased sensitivity to injury, ROS leakage, and reduced cellular ATP. Moreover, Sirt3 deacetylates transcription factor FoxO3a, thereby influencing SOD2 expression and further modulating ROS levels during ischemia-reperfusion in the heart [76, 78].

#### Autophagy and its regulators

Autophagy, a cellular housekeeping process involving the degradation and recycling of cellular components, plays a significant role in cardiac aging [79, 80]. With aging, both the generation and removal of autophagosomes decline [81], leading to premature cellular senescence [82]. Downregulation of autophagy in the heart may lead to premature cardiac aging [83, 84], while promoting autophagic flux is linked to anti-aging characteristics in mice [85]. Enhancing autophagy by autophagy enhancer torin1 or overexpressing ATG7 reduced the rate of ROS production and restored mitochondrial membrane potential and Ca^2+^-handling in cardiomyocytes from aged rabbit hearts [86].

Autophagy is negatively regulated by mammalian target of rapamycin (mTOR), a serine/threonine kinase, which operates through two complexes, mTORC1 and mTORC2. During food intake, mTORC1 is active and inhibits autophagy, while its inhibition during starvation reverses its inhibitory effect on autophagy. Insulin signaling inhibits autophagy by activation of PI3K/Akt/mTORC1 and repressing ATG gene expression. Attenuation of PI3K signaling in aged mice improved autophagic flux and reduced age-dependent decline in cardiac function [87]. Likewise, ablation of Akt2, a direct target of PI3K, in mice improved autophagy, preserved mitochondrial function, and protected against aging-induced cardiomyocyte dysfunction [88]. Moreover, old mice with low IGF1 receptor activity (and reduced PI3K/Akt signaling) or caloric restriction exhibited higher autophagic flux and improved myocardial bioenergetics [89-91]. In contrast, chronic Akt activation or forced mTORC1 activation in old mouse hearts during myocardial ischemia hampers autophagy and increases ischemic injury [92, 93]. Caloric restriction or nutrient deprivation activates AMPK, which stimulates autophagy via inhibition of mTORC1 signaling [94]. AMPK can also directly stimulate autophagy by phosphorylating ULK1 and relieving its inhibition from mTORC1 [95]. AMPK deficiency worsens cardiomyocyte function, intracellular Ca^2+^ handling, and mitochondrial health, leading to increased ROS production. Metformin (an activator of AMPK) treatment attenuates aging-induced contractile defects, highlighting AMPK's role in cardiac dysfunction during aging [96]. mTORC2, on the other hand, controls cell survival, insulin sensitivity, and cell polarity and influences autophagy indirectly via activation of Akt and mammalian sterile 20-like kinase 1 (Mst1) [97]. Mst1 activation triggered by pro-apoptotic stimuli, like ROS, phosphorylates and inactivates beclin1 and, therefore, inhibits autophagy [98]. Conversely, decreased Mst1 levels enhance autophagy, offering cardiomyocyte protection [81].

Sirtuins are important regulators of autophagy, and their activity is dependent on NAD^+^ availability. Thus, enzymes, like nicotinamide phosphoribosoyltransferase, increasing cellular NAD^+^ levels may directly or indirectly activate autophagy by modulating the activity of sirtuins [99-101]. Conversely, Sirt3 deficiency reduces autophagy flux and worsens cardiac hypertrophy, a condition that can be mitigated by overexpression of Sirt3 [102]. Similarly, Sirt6 overexpression activates FoxO3 by deacetylation, thereby enhancing autophagy and protecting against cardiac hypertrophy [103]. Additionally, Sirt6 overexpression has been shown to protect against doxorubicin-induced cardiomyocyte senescence [104]. In contrast, Sirt6 deficiency in cardiac fibroblasts leads to the activation of TGF-β signaling, which promotes myofibroblast transformation [105] and potentially contributing to the development of cardiac fibrosis. Likewise, Sirt6 deletion in bone marrow-derived cells results in hyperactivation of macrophages, leading to increased atherosclerosis [106].

Physiological levels of ROS are essential during ischemia- and nutrient deprivation-induced activation of autophagy in cardiomyocytes [107]. Depletion of physiological mitochondrial ROS by over-expression of catalase in mitochondria impairs the compensatory role in hearts leading to cardiomyopathy [108]. Similarly, the knockdown of NOX4 blunts autophagy flux and worsens ischemia-induced myocardial injury [107]. Conversely, excessive ROS production impairs cardiac autophagy [109], likely by oxidizing ATG3 and ATG7 [110].

### 1.3 Aging-related inflammation

#### Inflammaging

Inflammation serves as a physiological response of the immune system to pathogens, toxins, or tissue damage. While the resolution of the acute inflammation restores the immune homeostasis [111], a prolonged or persistent inflammatory state has damaging consequences [111, 112], contributing to the development of pathological conditions e.g., type II diabetes, metabolic syndrome, neurodegenerative diseases, cancer, and cardiovascular disease (CVD) [111-114].

Aging is often accompanied by chronic low-grade systemic inflammation, termed inflammaging [115], characterized by an increase in pro-inflammatory markers (e.g., CRP, TNF-α, IL-1β, IL-6, IL-8, and IL-18) [115-117] and an imbalance between pro-inflammatory and anti-inflammatory/pro-resolving cytokines [111, 118]. Inflammaging is closely associated with dysfunctional mitochondria, oxidative stress, inflammasome activation, DNA damage, and telomere shortening—molecular biomarkers of aging contributing to cellular senescence [119-123].

In dilated cardiomyopathy (DCM), aging appears to negatively influence the mitochondrial function and the pro-inflammatory/anti-inflammatory balance [124]. Moreover, disturbances in the inflammatory process are observed in older patients with DCM and inflammatory DCM (DCMI) with upregulation of IL-18 linked to DCMI, heart failure, and ischemic cardiac disease [125-127]. Elevated IL-6 [111, 128-131] and TNFα [132] expression are associated with aging and age-related chronic diseases such as CVDs, diabetes, and obesity [133, 134]. Lately, IL-12 is also associated with age-related CVDs [124], particularly in older male patients with DCM [124].

Recent data from our group have highlighted the role of sex in age-related inflammation. Higher vulnerability of older women with DCMI to immunometabolic changes and inflammatory imbalance at the cellular level has been observed [125]. Notably, estrogen loss has been implicated in the activation of pro-inflammatory pathways, reduction in anti-oxidative defense, and impaired mitochondrial homeostasis in the hearts of older females [17, 135-137]. Estrogen appears to influence the expression and activity of metabolic sensors such as Sirt1 and AMPK, as well as inflammatory responses in both in vivo and in vitro models [138-140].

While the causal relationship between inflammaging and aging remains unclear, inflammation is considered one of the seven mechanistic pillars of aging [141], along with proteostasis, adaptation to stress, metabolism, epigenetics, stem cells and regeneration, and macromolecular damage [141]. These interconnected processes are believed to underlie both the aging process and age-related diseases. Inflammaging, has been implicated not only in the development of various diseases common in older age [142] but also in cardiovascular conditions such as atherosclerosis and arterial stiffness, contributing to heart disease [143]. Additionally, certain diseases may accelerate aging processes—a phenomenon known as stress-induced premature senescence [141].

#### Immunosenescence

Immunosenescence, characterized by the age-related dysregulation of the immune system due to the permanent cell cycle arrest in immune cells [144], is a key driver of inflammaging [145]. Immunosenescence is induced by the age-related upregulation of NF-κB signaling driven by inflammation, activation of the innate immunity, tumor microenvironment, oxidative stress, DNA damage, or cytokine upregulation in immune cells and various tissues and in genetically modified mouse models [146-149]. Additionally, there are alterations in the function of toll-like receptors (TLRs) [150] and accumulation of senescent cells in various tissues, including the heart [151, 152]. Senescent cells produce a complex pro-inflammatory secretome known as a senescence-associated secretory phenotype (SASP) consisting of several pro-inflammatory cytokines, growth factors, proteases, chemokines, and matrix metalloproteasases [141, 153-156]. Additionally, senescence cells lead to increased production of ROS [157] causing DNA, protein, and lipid damage [158-160], thereby contributing to the pro-inflammatory response. Exacerbated ROS formation compromises mitochondrial function, promoting the development of several age-related diseases such as CVD [122, 123, 161]. This is further exacerbated by the decline in anti-oxidative defense with age, including enzymes like SOD2, catalase and glutathione peroxidase [111, 162], contributing to the perpetuation of inflammation [163]. Chronic oxidative stress may activate the NLRP3 inflammasome [164, 165], triggering prominent pro-inflammatory cascades via NF-κB including cytokines, chemokines, and adhesions molecules (e.g., TNF-α, IL-β, IL-6, IL-18, ICAM-1) [111, 166].

#### Inflammation and the metabolic sensors

The metabolic sensors AMPK and Sirt1 counteract the excessive inflammatory response by inhibiting NF-κB [167-169]. Specifically, Sirt1 negatively influences NF-κB activity through direct interaction with the RelA/p65 subunit of the NF-κB complex leading to deacetylation of lysine 310 at p65, resulting in the degradation of the p65 subunit [170]. Decreased AMPK activity is closely associated with increased inflammation [171], while AMPK activation inhibits inflammatory responses, such as NF-κB signaling [172, 173]. The AMPK-related NF-κB inhibition seems to be mediated via Forkhead box O (FoxO) or PGC-1α, which is a key regulator of mitochondrial function, oxidative stress, or inflammation [171, 174]. Conversely, inflammatory responses, including NF-κB p65 activation, suppress PGC-1α expression in cardiomyocytes, e.g., during hypoxia in primary rat cardiomyocytes [74, 175].

During aging, NF-κB expression and activity tend to increase, while Sirt1 and AMPK activity decline significantly [170]. Consistent with this, our data demonstrated reduced Sirt1 expression and AMPK activity in cardiac tissue from apparently healthy older women (without cardiac events), accompanied by an increased NF-κB expression [17]. In another study, we observed decreased Sirt1 expression and increased NF-κB expression in the hearts of male patients with idiopathic DCM [124]. In contrast, Kilic et al., reported an upregulation of plasma Sirt1 levels in elderly individuals compared to young children and adults [176], suggesting the activation of protective mechanisms during aging. However, the mechanisms by which circulating Sirt1 can provide protection against aging remain unclear and warrant further investigation.

AMPK activity appears to be affected by inflammatory cytokines. Indeed, AMPK activity declines in the presence of TNF-α via the upregulation of the AMPK inhibitor PP2C [177]. In mouse cardiac tissue, increased IL-6 levels correlate with a reduced AMPK activity, accompanied by reduced glucose uptake and a pro-inflammatory state in the heart [178]. Additionally, the upregulation of anti-inflammatory mediators (e.g., IL-10 and TGF-β) in the absence of pro-inflammatory mediators (e. g., IL-6) induces the phosphorylation and activation of AMPK in murine and human macrophages [179, 180].

**Figure 1.**
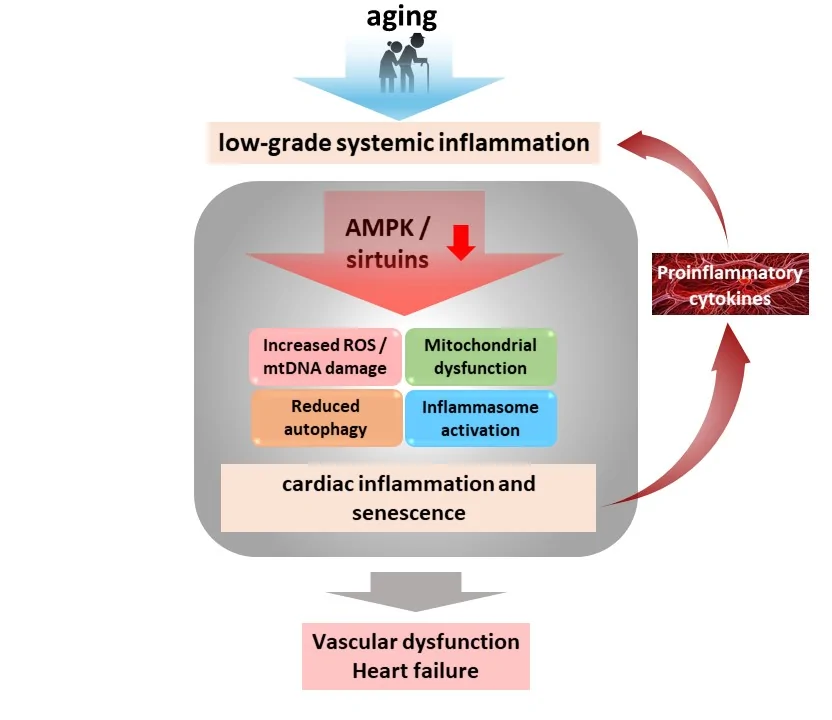
**Schematic presentation of the role of AMPK and sirtuins in cardiac aging**. Aging and the associated low-grade systemic inflammation lead to the reduced activity of cardiac AMPK and sirtuins. This causes a disturbance of mitochondrial and redox homeostasis, accompanied by a reduced autophagy and activation of inflammasomes. Overall, these events promote cardiac inflammation and senescence leading to the release of proinflammatory cytokines and further amplification of systemic inflammation. Finally, cardiac inflammation leads to vascular dysfunction and heart failure.

In summary, aging leads to the reduced activity of cardiac AMPK and sirtuins. This causes a disturbance of mitochondrial and redox homeostasis, accompanied by reduced autophagy and activation of inflammasomes that promote cardiac inflammation and senescence. This complex disturbance of cellular homeostasis consequently leads to vascular dysfunction and heart failure (Fig. 1).

## 2. Translational aspects

### 2.1 Heart failure

Heart failure (HF) is a syndrome associated with aging and is a significant contributor to morbidity and mortality in older individuals. There are two main types of left-side HF: systolic and diastolic HF. Systolic failure occurs due to decreased contractility of the left ventricle and is defined as a HF with reduced ejection (EF), or HFrEF when the EF<40%. Diastolic failure, on the other hand, results from the left ventricle’s inability to relax normally due to muscle stiffness. Thus, the heart cannot be properly filled with blood during the resting period between each beat and is characterized as HF with preserved EF, or (HFpEF), when the EF>50%.

#### Role of sirtuins in HF

Emerging evidence suggests that HF is accompanied by reduced activity of sirtuins leading to both cytosolic and mitochondrial protein hyperacetylation and impairment of metabolic control in cardiomyocytes. Indeed, a comprehensive study by Horton et al [181] using a mouse transverse aortic constriction (TAC) model of HF revealed that during the progression from compensated hypertrophy to HF, net mitochondrial protein acetylation increases. Similarly, cardiac samples from DCM patients show hyperacetylation of mitochondrial proteins. Notably, a substantial number of hyperacetylated proteins in HF samples are involved in key catabolic and ATP synthetic pathways, including fatty acid oxidation, TCA cycle, and mitochondrial ETC [181]. This hyperacetylation is accompanied by a significant reduction in NAD^+^ levels in cardiac samples obtained from TAC mice or DCM patients, suggesting decreased Sirt3 activity [181].

Interestingly, while both HFrEF and HFpEF exhibit protein hyperacetylation, the degree is more pronounced in HFpEF, correlating with reduced cellular NAD^+^ [182].

The cause for the NAD^+^ reduction in HF is still poorly understood but may involve, enhanced consumption by enzymes like CD38 [24] or by PARP [22], as well as reduced synthesis mediated by downregulation of Nampt, the rate-limiting enzyme in the NAD^+^ salvage pathway [183].

In addition to reduced sirtuin activity, HF is associated with downregulation of Sirt1 and Sirt3 expression in animal models [184, 185] and patients [186]. We recently demonstrated a significant downregulation of cardiac Sirt1 and Sirt3 in older, but not younger, patients with end-stage DCM [124]. However, in patients with DCMI induced by viral myocarditis, no alteration of Sirt1 or Sirt3 expression was observed [125]. Nevertheless, a significant hyperacetylation of mitochondrial proteins, indicated by SOD2 acetylation, in younger male patients with DCMI, whereas a significant deacetylation in older men has been observed. Therefore, there are marked age and sex effects in mitochondrial protein acetylation in patients with DCMI [125].

Protein hyperacetylation, resulting from reduced activity or expression of Sirt1 or Sirt3, contributes to impaired mitochondrial function and biogenesis [182], as well as increased inflammation [185]. However, a recent study by the Muoio group, using genetic deletion of Sirt3, found that elevated mitochondrial acetylation had only marginal effects on mitochondrial respiration [187]. In another study from the same group, a mouse model with deficiencies in both carnitine acetyltransferase and Sirt3, leading to hyperacetylation of mitochondrial proteins, showed a surprisingly normal bioenergetics profile [188]. Furthermore, hearts from these mice did not exhibit increased sensitivity to cardiac dysfunction in response to pressure overload. The cause of this discrepancy remains unclear but may be related to differences in the models used. Notably, a recent study by Hu et al. [189] did not observe a significant difference in cardiac function between Sirt3-KO and wild-type mice in the early stages, although Sirt3-KO mice exhibited a decline in cardiac function with increasing age.

Strategies to treat protein hyperacetylation in HF have focused on normalizing the NAD^+^/NADH ratio by supplementation with NAD^+^ precursors. Treatment with NAD^+^ precursor, nicotinamide riboside chloride for 4 to 8 weeks reduced protein acetylation, improved mitochondrial function, and ameliorated HFpEF phenotype in mice [182, 183]. Similarly, a recent study demonstrated the efficacy of NAD^+^ precursor nicotinamide in ameliorating diastolic dysfunction in different rodent models, including aging (2-year-old mice), hypertension (Dahl salt-sensitive rats), and cardiometabolic syndrome (ZDF rats) [190]. Additionally, *in vitro* treatment of peripheral blood mononuclear cells (PBMCs) isolated from HFrEF patients with nicotinamide riboside enhanced mitochondrial respiration and reduced pro-inflammatory cytokine production [191].

An alternative approach to restore cardiac NAD^+^ levels is the pharmacological activation of Nampt using P7C3-A20 [183]. Four weeks of treatment with P7C3-A20 restored cardiac NAD^+^ level and attenuated diastolic dysfunction in HFpEF hearts, further supporting the therapeutic potential of NAD^+^ repletion in HFpEF [183]. Clinical trials investigating the efficacy of NAD^+^ precursor supplementation in HF patients are currently ongoing with some preliminary evidence suggesting improvements in mitochondrial function and reduced inflammation in peripheral blood mononuclear cells [192]. However, clinical endpoints such as patients’ fitness, quality of life, and left ventricular function did not show significant improvement following nicotinamide riboside treatment [192]. Therefore, further research is needed to determine the impact of NAD^+^ precursor supplementation on clinical outcomes in HF patients. In this context, analyzing the upstream mechanisms underlying NAD^+^ depletion could provide valuable insights. One potential target is AMPK, which regulates substrate and energy metabolism, increases cellular NAD^+^ levels, and enhances Sirt1 activity in skeletal muscle [14].

#### Role of AMPK in HF

Contrary to the well-documented decrease in Sirt1 and Sirt3 expression and/or activity observed in HF, the impact of HF on AMPK is rather complex. AMPK, functioning as a stress-responsive kinase, is activated in response to various stressors like cardiac ischemia [193], ischemia-reperfusion injury [194], or chronic pressure overload [195]. However, during the progression of HFrEF or HFpEF, AMPK activity declines [196, 197] or remains unchanged [198]. This complexity is further compounded by isoform-specific dynamics in AMPK activity during HF development. Indeed, a comprehensive study employing the TAC mice model revealed an isoform switch from AMPKα2 to AMPKα1 in failing hearts [196]. While both AMPKα1 and AMPKα2 initially show increased expression and activity (5 days post-TAC), AMPKα2 expression begins to decline 14 days post-TAC, particularly when HFrEF is manifested, whereas AMPKα1 remained elevated. This isoform-specific alteration pattern was corroborated in patients (50 years old on average) with severe HFrEF, indicating increased expression and activity of AMPKα1 but reduced AMPKα2. Mechanistically, reduced phosphorylation of Ser495 in PINK1 by AMPKα2, crucial for efficient mitophagy, is implicated in promoting HF progression [196].

An inherent limitation of animal HF models is the absence of age and sex effects, despite the majority of HF patients being over 60 years old and HFpEF being twice as prevalent in women. Recently, we addressed the sex and age difference in AMPK activity and expression [124] in end-stage DCM patients. An elevation of AMPK expression and activity in younger and older men, but not in women was observed in DCMI patients due chronic myocarditis [125]. This elevation in AMPK was accompanied by enhanced autophagy and preserved mitochondrial biogenesis in older male DCMI patients, while autophagy was impaired in older female patients. Interestingly, a reduction in mitochondrial mass was observed in older patients regardless of sex, suggesting potential disruptions in mitochondrial homeostasis due to chronically elevated AMPK, particularly in older male patients, which may contribute to heart failure. This is supported by another study showing that activation of AMPK leads to phosphorylation of the mitochondrial fission factor resulting in mitochondrial fragmentation, a key event in mitochondrial clearance [199].

Sex and age differences in AMPK activity in HF appear to be contingent upon the type of HF. In contrast to the DCMI, in patients with idiopathic DCM, AMPK phosphorylation both in older male and female patients (>50 years) was elevated, whereas in younger patients (<40 years) was markedly reduced [124]. It can be inferred that aging-induced metabolic perturbations associated with AMPK downregulation in older patients [17, 200] may be exacerbated by HF, leading to compensatory activation of AMPK.

Furthermore, studies investigating HFpEF have reported a significant reduction in AMPK phosphorylation along with decreased expression of Sirt1, in animal models of HFpEF [197, 201] and pulmonary hypertension associated with HFpEF [202, 203]. Treatment with the sodium-dependent glucose transporter 2 (SGLT2) inhibitor canagliflozin, elevated AMPK activity and Sirt1 expression, leading to improvements in myocardial hypertrophy, fibrosis, and left ventricular diastolic dysfunction [201]. Although the molecular mechanisms underlying the effects of SGLT2 inhibitors on AMPK activity remain unclear, it is suggested that activation of sirtuins or suppression of mTOR may play a role [204]. Interestingly, SGLT2 inhibition has been shown to eliminate senescent cells and alleviate aging phenotypes in mice through AMPK activation [205], suggesting a potential therapeutic approach for treating cardiac aging-related pathologies in humans.

In summary, HF is intricately associated with reduced expression and activity of AMPK and sirtuins, which exacerbate the age-induced downregulation of these metabolic sensors. Further elucidation of the mechanisms underlying AMPK dysregulation in HF and exploration of potential therapeutic strategies targeting AMPK and sirtuins pathways are warranted.

### 2.2 Vascular pathologies

Vascular aging correlates with increased atherosclerosis and microvascular dysfunction [206, 207], manifesting as pathological vascular remodeling and stiffness [208]. Key contributors to vascular stiffening include fibrosis, perivascular inflammation, and vascular calcification [209]. Senescence of both endothelial and vascular smooth muscle cells (VSMCs), characterized by replicative and stress-induced premature senescence, plays a pivotal role in the development of atherosclerosis [210, 211]. These senescent cells exhibit morphological and gene expression changes, culminating in impaired vascular function, inflammation, thrombosis, and atherosclerosis [212]. Evidence suggests that vascular aging begins early in life, featuring progressive alterations in vascular structure and function, ultimately leading to decreased vascular compliance and increased arterial stiffness [213]. Impaired endothelium-dependent vasodilation and reduced vessel repair capacity are hallmarks of vascular aging [214]. Both sirtuins and AMPK play crucial roles individually and in coordination in regulating various aspects of vascular function. These include but not limited to endothelial function [215], angiogenesis [216, 217], endothelial senescence [218-220], endothelial metabolism [221, 222], and vascular tone [223-225]. Dysregulation of the expression and activity of sirtuins and AMPK in vascular cells can lead to endothelial dysfunction, which is implicated in the development of aging-related various vascular pathologies such as atherosclerosis [222, 226-232]. Cellular and molecular factors contributing to vascular aging are discussed below:

*ROS mediated oxidative stress* drives vascular aging, fueled by unregulated ROS production from NADPH oxidases [233, 234] and dysfunctional mitochondria [235] and contribute to endothelial dysfunction and arterial stiffening. Consequently, bioavailability of endothelium-derived NO is inactivated, reducing endothelium-dependent dilation, increasing vascular inflammation, and promoting pro-atherogenic vascular changes. Increased ROS generation in young organisms activates Nrf2 pathway, reducing oxidative stress and displaying anti-inflammatory effects [236]. However, aging disrupts Nrf2, exacerbating oxidative stress and vulnerability to vascular damage. Activation of AMPK and sirtuins through strategies such as caloric restriction or pharmacological activation of AMPK and sirtuins induces Nrf2, suggesting potential anti-aging benefits [220, 237-240]. Further investigation is needed for pharmacological Nrf2 activation to achieve anti-aging vasoprotective benefits.

*Mitochondrial dysfunction*, characterized by decreased respiratory chain efficiency and increased ROS production, is central in aging and vascular aging [65, 241, 242]. Dysfunctional mitochondria, impaired energy metabolism, and mtDNA damage contribute to age-related vascular dysfunction, exacerbated by impaired biogenesis and mitophagy [242]. Increased mitochondrial ROS production, resulting from a dysfunctional ETC and impaired antioxidant responses further aggravates vascular dysfunction [243-245]. Targeting mitochondrial ROS with agents activating AMPK such as MitoQ and resveratrol improves endothelial function [246, 247] and ameliorate atherosclerosis [248]. Sirt1 and Sirt3 are key regulators of mitochondrial function, and strategies enhancing NAD^+^ biosynthesis or pharmacological activation of Sirt1 or Sirt3 hold potential for reversing age-related vascular changes [22, 231, 237, 249-251].

*Inflammaging* is a characteristic feature of vascular aging, contributing to various vascular problems (for detailed review see [119]). This pro-inflammatory microenvironment in blood vessels leads to dysfunction, metabolic impairment, apoptosis, and contributes to various vascular diseases, including atherogenesis, aneurysm formation, and Alzheimer's pathologies [252]. Approaches towards the activation of the sirtuins/AMPK pathway hold considerable potential in mitigating inflammaging, as evidenced by recent findings [253].

*Proteostasis* is vital for the proper protein folding and degradation and its impairment contributes to vascular aging [144]. Age-related imbalances between protein synthesis, maintenance, and degradation lead to increased misfolded protein aggregates, linked to vascular pathologies. Aging negatively affects the activity of proteostasis components like chaperones, the ubiquitin-proteasome system, and lysosome-autophagy processes [254-257]. Chaperones prevent misfolding, but their activity declines with age, while dysregulated autophagy, including mitophagy, may exacerbate vascular aging [252, 258]. There is growing evidence indicating the involvement of sirtuins and AMPK signaling in proteostasis in various contexts such as cellular homeostasis and aging, however, the specific relationship between AMPK, sirtuins, and vascular proteostasis remains understudied. Future research should explore age-related factors regulating proteostasis pathways in the vascular wall and the potential roles of AMPK and sirtuins in maintaining vascular proteostasis and how dysregulation of these pathways may contribute to vascular aging and associated pathologies [259, 260].

*Cellular senescence* is a key aging process where cells, including ECs and VSMCs, cease dividing due to factors like oxidative stress, DNA damage, and paracrine signals [261]. EC senescence contributes to endothelial dysfunction and impaired regenerative capacity [262-264]. Senescent cells present in atherosclerotic lesions and may promote plaque instability and vascular inflammation. Eliminating senescent cells with senolytics holds the potential for atheroprotective effects and improving endothelial function [265-267]. However, further exploration is needed to understand the precise role of different senescence mechanisms in age-related vascular diseases [261]. Sirt1 and LKB1/AMPK pathways coordinately regulate cell survival, proliferation, and senescence. The LKB1/AMPK pathway allows cells to adapt to stress when activated acutely [268-270], but chronic activation leads to growth arrest and senescence [219, 271, 272]. Sirt1 fine-tunes the LKB1/AMPK pathway and prevents it from shifting from a pro-survival to a pro-aging proliferation arrest state [219]. Further research on the regulatory mechanisms and pathways affecting the balance between Sirt1 and LKB1/AMPK could provide significant insights into controlling the aging process.

*Cellular energy-sensing pathways*, including mTOR, AMPK, and sirtuins, regulate aging processes in response to nutrient availability and growth signals [273-275]. Inhibiting mTOR extends lifespan and delays age-related diseases [276-278] also offering vascular protection by delaying EC senescence and promoting vasodilation. Sirtuins and AMPK, activated by metabolic factors and interventions like caloric restriction [279-281], have diverse anti-aging effects in the vasculature, improving endothelial function, reducing oxidative stress, and exerting anti-inflammatory and anti-atherogenic effects [238, 281]. Compounds activating these pathways, such as resveratrol, NAD^+^, MDL-800 (Sirt6 activator), and metformin, are potential drug targets for cardiovascular protection in older individuals [282-287].

### 2.3 Translational aspects of inflammaging

Several studies have demonstrated the ability of metformin, an AMPK activator, to suppress systemic inflammation in patients with metabolic syndrome as evidenced by reductions in IL-6 and CRP levels [288]. Targeting AMPK and its agonists represents a promising avenue for addressing cardiac inflammaging. For instance, the AMPK agonist, A-769662, which specifically activates the AMPK β1 subunit, attenuates the expression of the pro-inflammatory marker IL-6 in inflammatory arthritis [289]. Moreover, A769662 restores the AMPK activity and autophagy via IL-6 inhibition in inflammatory disorders [290]. Additionally, telmisartan, an angiotensin II receptor antagonist, indirectly activates AMPK via PPARγ in skeletal muscle cells in obese db/db mice [291].

Metformin, a widely studied drug, has been tested in large clinical trials to treat age-related disorders and improve healthspan. The therapeutic effects of metformin are related to the inhibition of mitochondrial complex I, leading to an increase in cytoplasmatic AMP:ATP and ADP:ATP ratios and subsequent AMPK activation [292]. This mechanism is associated with a reduction of oxidative stress and the production of several pro-inflammatory cytokines (e.g. IL6 and IL-8) [292, 293]. Other AMPK agonists, investigated in several clinical trials include nutraceuticals like resveratrol, and appears to ameliorate inflammaging [54, 119, 294].

Various interventions, such as dietary, pharmacological (e.g., rapamycin, metformin, or acarbose), or genetic (e.g., SLO-1 knockdown, mTORC1 knockdown, Sirt1 overexpression), have been developed to prolong lifespan and extend healthspan [295-299]. Notably, centenarians and nonagenarians, who age more slowly and enjoy a prolonged healthspan [111], serve as noteworthy examples, with the Mediterranean diet potentially contributing to this phenomenon [111]. Additionally, caloric restriction has demonstrated positive effects on lifespan in worms and mice [300-302], although its relevance in human aging remains unclear. The interplay between aging and diet/fasting appears to profoundly impact inflammation and chronic age-related diseases [303]. In this context, both caloric restriction and exercise show positive effects on modulating the pro-inflammatory profile, reducing disease symptoms, and promoting autophagy in in vivo aging models [304, 305]. Rapamycin and its analogs are currently under investigation for their potential therapeutic use in various age-related diseases (for a detailed review, see Mannick and Lamming) [306]. However, these agents are associated with severe side effects, including diabetic-like insulin resistance, male infertility, and immune-suppression.

In conclusion, cardiac inflammaging promotes metabolic imbalance, mitochondrial dysfunction, dysfunctional microbiome, and vascular senescence. Therapeutic interventions targeting inflammaging hold promise for preventing cardiac aging and age-related CVDs.

### 2.4 Gut microbiome

#### Gut microbiota and aging

The human gut microbiome undergoes rapid changes in the early years of life, maintaining consistency thereafter, but age-related alterations become evident in older adults (65+ years) [307, 308]. Clinical studies show significant difference in microbiome composition between young and elderly individuals [309-311], with decreased diversity observed in the elderly. Reduced microbiome diversity may contribute to the development of various age-related diseases like colitis [312], type 1 diabetes [313, 314], and rheumatoid arthritis [315-317], although the direct impact on aging remains unclear. The gut hypothesis suggests that increased systemic congestion and decreased cardiac output may cause edema and ischemia of the gut mucosa that leads to bacterial translocation into the blood and increased circulating endotoxin, contributing to HF [318, 319]. Clinical evidence indicates that post-myocardial infarction (MI) cardiovascular outcomes are influenced by gut microbiota translocation, with gut-derived bacteria and metabolites correlating with systemic inflammation. Likewise, animal studies suggest that preserving gut barrier integrity could mitigate cardiovascular events post-MI, highlighting potential therapeutic strategies [320].

Overall, aging is associated with an increase in *Enterobacteriaceae* [321] and a decrease in *Lactobacillus* and *Bifidobacterium* levels [322], often influenced by dietary changes [323, 324], lifestyle [325], a weakened immune system [326], medications [327], infections [328], and decreased intestinal functionality [325, 329, 330]. Interestingly, in centenarians and supercentenarians, *Bifidobacteria* and *Christensenella* are particularly abundant [331, 332].

#### Impact of aging on gut microbiota composition

During aging, the ratio between *Firmicutes* (F, Gram-positive), important for short chain fatty acids (SCFAs) production, and *Bacteroidetes* (B, Gram-negative), vital for polysaccharide metabolism, increases from birth to adulthood and decreases in the elderly. A study by Mariat et al. comparing human fecal microbiota across three age groups- infants (3 weeks to 10 months), adults (25 to 45 years), and elderly (70 to 90 years) - revealed an altered intestinal microbiota from birth to adulthood and again from adulthood to the elderly. The F/B ratios for infants, adults, and elderly of 0.4, 10.9, and 0.6 reflected the age-related changes [333].

Diets high in fiber, increase SCFA acid-producing bacteria, protect against pathogenic bacteria, lower blood pressure, reduce cardiac fibrosis and hypertrophy, and improve insulin sensitivity [334-337]. In addition, while intestinal SCFA decreased in the elderly, specific butyrate-producing bacteria have been detected in centenarians [338, 339]. Thereby a rearrangement of butyrate-producing bacteria was observed. While species of *Clostridium cluster XIVa*, and *Papillibacter cinnamovorans*, and *Clostridium cluster IV* decreased, species of *Clostridium cluster IV* and *XV* increased. Particularly, Eubacterium limosum (Clostridium cluster XV) was 15-fold higher as in other age groups and possibly indicates a group of bacteria, characteristic of long life [340]. In addition, SCFA improved intestinal barrier function and AMPK activity [341]. Peng et al. demonstrated that butyrate increased AMPK activity and reorganization of tight junction proteins. In addition, AMPK was upregulated upon calcium-mediated tight junction assembly. Using AMPK inhibitor compound C accelerated assembly of tight junctions and improved transepithelial electrical resistance was abolished, demonstrating the role of AMPK in the development of the intestinal barrier.

Furthermore, a study by Wilmanski et al. showed that in healthy elderly, increasing compositional uniqueness predicted survival in older adults [342]. In their study healthy individuals >80 years showed a microbial drift towards a more homogenous composition of microbiota, whereas less healthy individuals did not. By analyzing the microbiome of both groups, *Bacteroidetes* were depleted in the healthy aging elderly. It was concluded that microbiota uniformity is a component of healthy aging, characterized by metabolites in the blood, such as phenylacetylglutamine, also enriched in centenarians [343]. Identified microbiota pattern of healthy individuals, classified by medication use, self-perceived health, life-space score, walking speed, was marked by a decrease in *Bacteroides* which requires further investigation.

#### Impact of gut microbiota metabolites on development of CVD

Microbial sequencing analysis revealed information about the presence of distinct bacteria associated with metabolic disturbances [344] and CVD [345, 346]. Gut microbiota of patients with type 2 diabetes and insulin resistance produced higher levels of imidazole propionate (ImP) from histidine [347]. ImP was associated with HFrEF in two large clinical cohorts, independent of traditional CVD risk factors, such as age, sex, BMI, smoking status, systolic blood pressure, ethnicity, use of statins, diabetes status or circulating HDL, LDL and triglycerides [347]. Metagenomic sequencing analyses revealed that microbiota of patients with atherosclerotic CVD differed from those of healthy individuals, including higher levels of *Streptococcus* and *Enterobacteriaceae* [348]. Besides ImP, several other metabolites, including LPS and trimethylamine N-oxide (TMAO), can exert pro-atherosclerotic and -inflammatory effects promoting the development of CVDs and are outlined within the next sub-sections.

#### LPS in the development of CVD

Altered composition, function and metabolites of microbiota can be influenced by genetics [349-351], aging [342], and environmental factors such as diet [352], lifestyle or pollutants [353, 354]. In particular, LPS, structural components in the outer membrane of Gram-negative bacteria acting as bacterial toxins, reduced intestinal function. They impaired glucose metabolism and led to insulin resistance, thereby increasing the risk for CVD [355]. In addition, plasma LPS levels correlated positively with hypertension [356]. LPS-mediated intestinal bacterial dysbiosis and increased oxidative stress inhibited expression of endothelial nitric oxide synthase (eNOS) and NO production, further exacerbating hypertension through vasoconstriction. LPS also decreased the phosphorylation of AMPK and its target protein acetyl-CoA carboxylase in a dose-dependent manner [290]. Interestingly, activation of AMPK might directly impact the composition of microbiota as obese and diabetic patients taking metformin showed altered gut microbiome [357, 358]. Consistently AMPKα1^fl/fl^ mice treated with metformin also showed an alteration in gut microbiome [359]. Noteworthy, the gut microbiota profile of intestinal epithelium-specific AMPKα1-IKO mice was markedly shifted, displaying significantly higher abundance of *Mycoplasmataceae* and *Bacteroides* families, while abundance of beneficial *Lachnospiraceae* was significantly lower, compared to AMPKα1^fl/fl^ controls. This suggests that bacteria known to modulate lipid, glucose and energy metabolism are highly abundant in AMPKα1-KO mice, potentially compensating specific functions of intestinal AMPK. Moreover, levels of *Lachnospiraceae* in wildtype mice could be induced upon cold stimulation, suggesting a role in intestinal AMPK-mediated energy expenditure [359]. Thus, the shift in bacterial genera occurred in an intestinal AMPK-dependent manner, indicating that the metformin-mediated alterations of gut microbiota observed in previous studies may rely on changes in intestinal AMPK. In intestines of AMPKα1^fl/fl^ mice metformin induced Reg3γ expression, an antibacterial lectin produced by paneth cells that balance mucus distribution and are associated with anti-inflammatory macrophages and *Lactobacillus NK318.1* [360]. In contrast, Reg3γ expression in AMPKα1-IKO mice was not altered by metformin, suggesting a direct link to AMPK. One mechanism could be the AMP-induced change in intestinal Reg3γ expression due to the loss of active AMPK. Reg3γ has a bactericidal effect preferentially against Gram-positive bacteria and influences mucus distribution [361-363]. Thus, impaired Reg3γ expression in AMPKα1-IKO mice can alter the microbiome profile and metabolic products via AMP-AMPK-Reg3γ axis.

#### TMAO in the development of CVD

Trimethylamine N-oxide (TMAO), a key metabolite linked to gut microbiota, impacts cholesterol metabolism, oxidative stress, immune system regulation and inflammation, thereby increasing the risk of CVDs [364-366]. Dietary nutrients, such as choline, L-carnitine and phosphatidylcholine mainly present in red meat, eggs and fish, are metabolized by gut microbes into trimethylamine, which is converted to TMAO in the liver. TMAO is released into the circulation, potentially contributing to the promotion of atherosclerosis [367]. High TMAO levels are associated with coronary plaques, MI, myocardial hypertrophy, mitochondrial dysfunction, fibrosis, and CVD severity [368-372]. Moreover, TMAO triggers inflammation and is related to an increased risk of heart attack and stroke [373, 374], independent of other risk factors like triglycerides, blood pressure, and cholesterol.

Besides activation of NF-κB pathway in ECs and VSMCs [375, 376], TMAO can induce the NLRP3-inflammasome by inhibiting the Sirt3-SOD2-pathway. Sirt3 can bind and acetylate SOD2 directly, increasing SOD2 activity and thereby regulating mitochondrial ROS and NLRP3 inflammasome activation. Consequently, TMAO promotes vascular inflammation through the inhibition of Sirt3-SOD2 signaling [377]. Inflammatory pathways are particularly relevant in atrial fibrillation (AF), as they contribute to disease susceptibility. Metagenomic and metabolomic analyses on samples of AF patients revealed that dysbiotic gut microbiota appear in the early phase of AF and is likely an early regulator of disease progression [378]. A study by Luciani et al. showed that high levels of TMAO in AF patients were associated with increased risk of cardiovascular mortality and cerebral infarction [379]. Despite TMAO’s crucial role in pathogenesis of AF, further studies are needed to verify its regulatory effects in AF, for example, by targeting TMAO regulation independently of AF and/or in AF animal models with induced high TMAO serum levels [380].

Additionally, TMAO treatment increased cardiomyocytes size, atrial natriuretic peptide (ANP) and β-myosin heavy chain (β-MHC) levels, while a decrease in TMAO level with antibiotic therapy significantly reduced cardiac hypertrophy [381]. TMAO also increased total ROS levels and decreased Sirt1 and AMPK expression in an *in vitro* vascular cell model [382] and may have a role in cellular senescence [383].
